# Functional Characterization of Grapevine *VviMYC4* in Regulating Drought Tolerance by Mediating Flavonol Biosynthesis

**DOI:** 10.3390/plants14101409

**Published:** 2025-05-08

**Authors:** Yiting Tan, Wenjuan Wang, Wenbo Tian, Beibei Wang, Qifeng Zhao, Jinjun Liang, Wei Zhao, Pengfei Wen

**Affiliations:** 1 College of Horticulture, Shanxi Agricultural University, Taigu 030801, China; tyt_vitis@163.com (Y.T.); wwj619a@163.com (W.W.); 18234411280@163.com (W.T.); w0504839@163.com (B.W.); liangjinjun1989@163.com (J.L.); 2 Shanxi Key Laboratory of Germplasm Improvement and Utilization in Pomology, Pomology Institute, Shanxi Agricultural University, Taiyuan 030031, China; gsskyb@126.com

**Keywords:** grapevine, drought stress, flavonol biosynthesis, gene function, transcriptional regulation

## Abstract

Drought ranks among the key abiotic stresses that limit the growth and yield of grapevines (*Vitis vinifera* L.). Flavonols, a class of antioxidants commonly found in grapevines, play a crucial role in combating drought stress. In this study, we characterized the function and regulatory mechanism of the grapevine *VviMYC4* in mediating flavonol biosynthesis in response to drought stress. *VviMYC4* encodes a protein of 468 amino acids with conserved bHLH-MYC_N and bHLH domains. Phylogenetic analysis confirmed its homology with the grapevine VviMYC2 and similarity in function. The expression of *VviMYC4* in ‘Cabernet Sauvignon’ grapevine seedling leaves increased initially and then decreased during prolonged drought stress. The homologous and heterologous transformation of *VviMYC4* in grape suspension cells, *Arabidopsis* plants, tobacco leaves, and grapevine leaves demonstrated its ability to positively regulate flavonol biosynthesis and accumulation by promoting the expression of flavonol-related genes, thereby enhancing the drought tolerance of transgenic plants. Furthermore, VviMYC4 could bind to specific E-box sites on the promoters of *VviF3H* and *VviFLS* to improve their activities. This study highlights *VviMYC4* as a pivotal positive regulator of drought tolerance in grapevines and proposes that *VviMYC4* enhances the antioxidant and reactive oxygen species (ROS) scavenging abilities of grapevines in challenging environments and improves their stress resilience by mediating flavonol biosynthesis. Our findings offer crucial candidate genes and valuable insights for the molecular breeding of grapevine drought resistance.

## 1. Introduction

As an essential ecological factor in the growth, development, and reproduction of plants, water directly impacts the entire life cycle of plants from vegetative growth to reproductive development [1]. Under global warming, the drought stress caused by water resource shortage has become an important environmental limiting factor that restricts the sustainable development of modern agriculture and affects crop yield formation and quality improvement [2]. Throughout the long course of evolution, plants have developed multi-dimensional coping mechanisms to adapt to the environment of drought stress [3]. By perceiving and transmitting drought signals, plants respond systematically in terms of their morphological characteristics, physiological metabolic activities, hormone regulation networks, proteomic changes, and gene expression regulation to achieve adaptive regulation in the drought environment [4]. With the development of the modern biotechnology field, significant progress has been made in the research on plant drought tolerance mechanisms. This breakthrough has prompted the transformation of drought-tolerant crop breeding strategies from conventional breeding to molecular-level breeding [5]. From the perspective of molecular biology, drought stress can induce changes in the expression profiles of a large number of genes, including key genes involved in the stress signal transduction and transcriptional regulation of a large number of functional proteins. These genes together constitute the molecular regulatory network of plant drought stress resistance [6,7]. Revealing the expression regulatory network and its molecular mechanism of plant drought-resistance-related genes through gene isolation and functional identification techniques is the key prerequisite for using modern biotechnology to improve the drought resistance of crops [8].

Flavonoids, a group of secondary metabolites produced by plants, play a protective role in harsh environmental conditions [9,10]. Among them, flavonols stand out due to their potent antioxidant properties, which enable them to enhance the antioxidant enzyme activity and neutralize the overabundance of reactive oxygen species (ROS) triggered by drought conditions [11]. Numerous studies have revealed that when plants are subjected to water-deficient conditions, the key regulatory components within the flavonol metabolic pathway exhibit a marked upregulation. Concurrently, there is a notable increase in the biosynthesis of flavonol-derived secondary metabolites. This cascade of physiological and biochemical responses underscores their significant role in helping plants resist and adapt to drought stress [12,13]. The grapevine *VvbHLH1* promotes the biosynthesis and accumulation of flavonols and other flavonoids, thereby improving the drought tolerance of transgenic *A. thaliana* plants [14]. Meanwhile, overexpression of key flavonol biosynthesis genes, *DoFLS1* from *Dendrobium officinale* and *EkFLS* from *Euphorbia kansui*, could enhance the flavonol accumulation and drought resistance in *A. thaliana* [15,16]. Furthermore, flavonols have been established to modulate the stomatal movement influenced by hydrogen peroxide (H_2_O_2_) [7,17].

Transcription factors (TFs), being key players in gene expression regulation, can directly act on the structural genes in the flavonol biosynthesis pathway, regulating their expression levels and thereby influencing the metabolic processes of flavonols [18]. Myelocytomatosis (MYC) TFs belong to the basic helix–loop–helix (bHLH) superfamily, characterized not only by the presence of a bHLH domain but also by a conserved bHLH-MYC_N domain at the N-terminus. This domain is key to recognizing and binding to the promoter regions of target genes, thereby regulating their expression [19]. Facing the challenges of abiotic stress, the expression levels of key enzyme genes in the flavonol metabolic pathway, such as flavanone-3-hydroxylase (F3H) and flavonol synthase (FLS), can be precisely regulated by MYC transcription factors at the transcriptional level. This regulatory mechanism enhances flavonol biosynthesis within plants, equipping them to better withstand environmental challenges [20]. Our prior study has confirmed that VviMYC2 specifically binds to the promoter of the *VviFLS5* gene, regulating flavonol biosynthesis in response to drought stress in ‘Cabernet Sauvignon’ grapevine seedlings [21]. Limited relevant research on other species allows the above results to offer valuable insights for investigating the functional characterization and regulatory networks of MYC transcription factors across various plants.

Grapevines (*Vitis vinifera* L.), one of the vital cash crops, play a pivotal role in both agricultural production and consumer markets due to their rich flavonoid content and remarkable ecological adaptability [22]. However, persistent drought stress not only severely hampers the physiological development of grapevines and diminishes fruit quality but also poses significant challenges to the sustainable growth and economic viability of the industry [23]. In light of this, clarifying the regulatory mechanism of grapevines’ response to drought stress at the molecular level and then enhancing their drought resistance capacity have important theoretical value and practical significance for promoting grapevine stress resistance breeding, variety structure upgrading, and high-quality industrial development. Our preliminary research foundation indicated that the *VviMYC4* gene (LOC100246135) is significantly induced by exogenous methyl jasmonate under drought stress and positively correlates with flavonol accumulation in ‘Cabernet Sauvignon’ grapevine seedlings. Here, we confirmed that there is significant sequence homology between VviMYC4 and VviMYC2. Further study revealed that *VviMYC4* could directly respond to drought stress and specifically activate the expression of the *VviF3H* and *VviFLS* genes to mediate flavonol biosynthesis and thus enhance the grapevine’s ability to withstand drought stress. Elucidating the role of *VviMYC4* can enrich our understanding of the molecular regulatory network of grapevines under drought stress and provide a crucial avenue for developing strategies to enhance drought resistance and biomass accumulation.

## 2. Results

### 2.1. Molecular Cloning and Characterization of VviMYC4 in Grapevine

**Figure 1:**
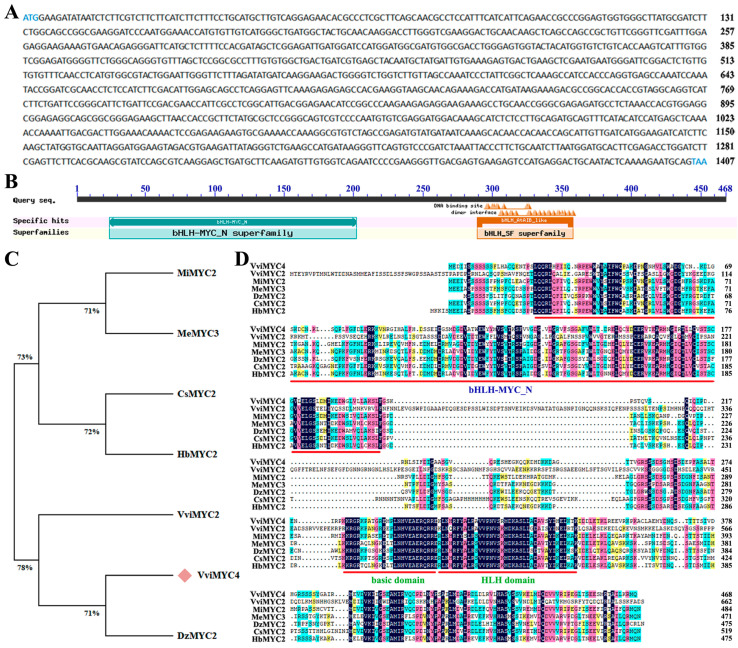
The bioinformatic analysis of VviMYC4. (**A**) The CDS full-length sequence of the *VviMYC4* gene in the ‘Cabernet Sauvignon’ grapevine. (**B**) Conserved domain prediction for the VviMYC4 protein. (**C**) Phylogenetic tree analysis of VviMYC4 and other MYC proteins. *Mangifera indica* MiMYC2, XP_044484728.1; *Manihot esculenta* MeMYC3, XP_021603159.1; *Citrus sinensis* CsMYC2, KAH9774897.1; *Hevea brasiliensis* HbMYC2; *Vitis vinifera* VviMYC2, XP_002280253.3; *Vitis vinifera* VviMYC4, XP_002279973.1; *Durio zibethinus* DzMYC2, XP_022738037.1. (**D**) Multiple sequence alignment of the MYC proteins in different species. Dark blue indicates 100% identity of amino acids, pink indicates 75% identity of amino acids, light blue indicates 50% identity of amino acids, and yellow indicates 33% identity of amino acids.

The *VviMYC4* gene was isolated from the cDNA of ‘Cabernet Sauvignon’ grapevine leaves. The coding sequence (CDS) of the *VviMYC4* gene is 1407 bp long and encodes 468 amino acids (Figure 1A). Conservative domain analysis showed that the protein contains two conserved domains, including bHLH-MYC-N- and bHLH_AtAIB-like domains (Figure 1B). Phylogenetic analysis highlighted that VviMYC4 shares the closest genetic resemblance with VviMYC2 from *Vitis vinifera* and DzMYC2 from *Durio zibethinus* (Figure 1C). Multiple sequence alignment further verified that the structure of the VviMYC4 protein is in line with the MYC proteins in other species, and they both belong to typical bHLH-MYC transcription factors (Figure 1D).

### 2.2. Expression Analysis of VviMYC4 in ‘Cabernet Sauvignon’ Grapevine Leaves Under Drought Stress

**Figure 2:**
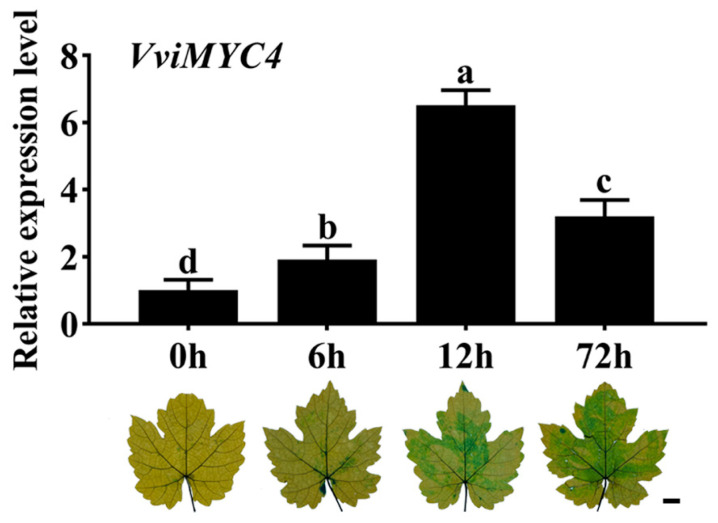
Expression profile of *VviMYC4* in response to drought stress in ‘Cabernet Sauvignon’ grapevine leaves. Different lowercase letters indicate the significance of the differences between samples (*p* ≤ 0.05). Leaves of ‘Cabernet Sauvignon’ grapevine were utilized for Evans blue staining to assess the cell viability. Bar = 1 cm.

To assess the responsiveness of the *VviMYC4* gene to drought stress, qRT-PCR analysis was employed to measure its expression in ‘Cabernet Sauvignon’ grapevine leaves across various drought exposure phases. The results indicated that the expression of *VviMYC4* was significantly induced by drought stress, showing a trend of first increasing and then decreasing within 72 h, reaching a peak at 12 h. Evans blue staining of grapevine leaves demonstrated the cell activity at different durations of drought treatment. As time went by, the activity of the leaf cells decreased, and the leaves gradually showed a deeper blue color (Figure 2).

### 2.3. Functional Analysis of VviMYC4 in Enhancing Drought Tolerance of Transgenic Grape Suspension Cells

**Figure 3:**
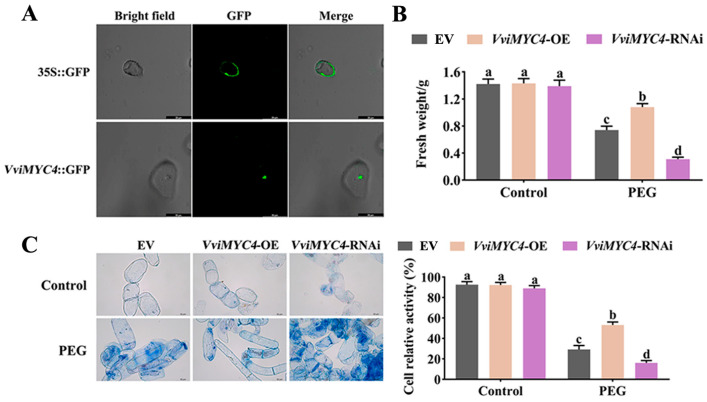
The performance of *VviMYC4* transgenic grape suspension cells under drought stress. (**A**) Fluorescence images of the empty vector and *VviMYC4* transgenic grape suspension cells. Green indicates fluorescence signal. (**B**) Fresh weight of the *VviMYC4* transgenic grape suspension cells under normal and drought conditions. (**C**) Evans blue stained images and cell relative activity of the *VviMYC4* transgenic grape suspension cells under normal and drought conditions. Bars = 50 μm. Blue indicates the degree of cell membrane damage. Different lowercase letters indicate the significance of the differences between samples (*p* ≤ 0.05).

To investigate the drought resistance function of *VviMYC4* in grapevines, vectors for overexpressing (OE) and RNA interfering (RNAi) *VviMYC4* with the 35S promoter were constructed and used to transform grape suspension cells, and positive transgenic cells were identified by GFP fluorescence. As shown in Figure 3, the transgenic suspension cells with the empty vector showed green fluorescence throughout the cells, while *VviMYC4*-GFP showed green fluorescence only in the nucleus. Further, the drought resistance was evaluated by measuring the fresh weight of the transgenic grape suspension cells. Under normal conditions, there were no obvious differences in the fresh weight of the different suspension cell types, while PEG treatment led to a significant decrease in the fresh weight of both the EV and transgenic suspension cells. The decline rate of the *VviMYC4*-OE cell content was lower than that of the EV cells, while the decline proportion of the *VviMYC4*-RNAi suspension cell content was higher (Figure 3B). The Evans blue results of the suspension cells showed that compared with EV, there were fewer blue cells in *VviMYC4*-OE and more blue cells in *VviMYC4*-RNAi, consistent with the cell relative activity measurements (Figure 3C). The above results indicate that *VviMYC4* can significantly enhance the drought tolerance of grape suspension cells.

### 2.4. Functional Analysis of VviMYC4 in Mediating Flavonol Biosynthesis to Enhance Drought Tolerance in Transgenic A. thaliana

**Figure 4:**
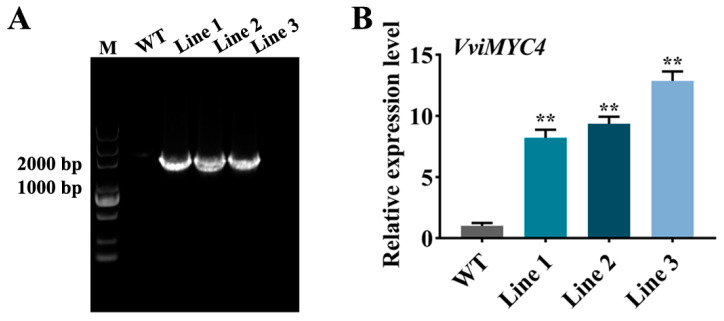
PCR detection results and gene expression analysis of the *VviMYC4* transgenic *A. thaliana* positive plants. (**A**) PCR detection results of the *VviMYC4* transgenic *A. thaliana* plants. Marker, DL5000. WT, the negative control with wild-type *A. thaliana* as the template. Lines 1–3, transgenic *A. thaliana* plants. (**B**) *VviMYC4* gene expression analysis in the transgenic *A. thaliana* plants. ** indicate the significant differences between the different transgenic lines and wild-type lines (*p* ≤ 0.01).

To further demonstrate the role of the *VviMYC4* gene in mediating flavonol biosynthesis to regulate drought resistance, transgenic validation was performed in *A. thaliana*. Based on the PCR detection and RT-qPCR gene expression analysis, three homozygous transgenic *A. thaliana* lines, Lines 1/2/3, with significantly higher *VviMYC4* expression levels were selected for further analysis (Figure 4A,B).

**Figure 5:**
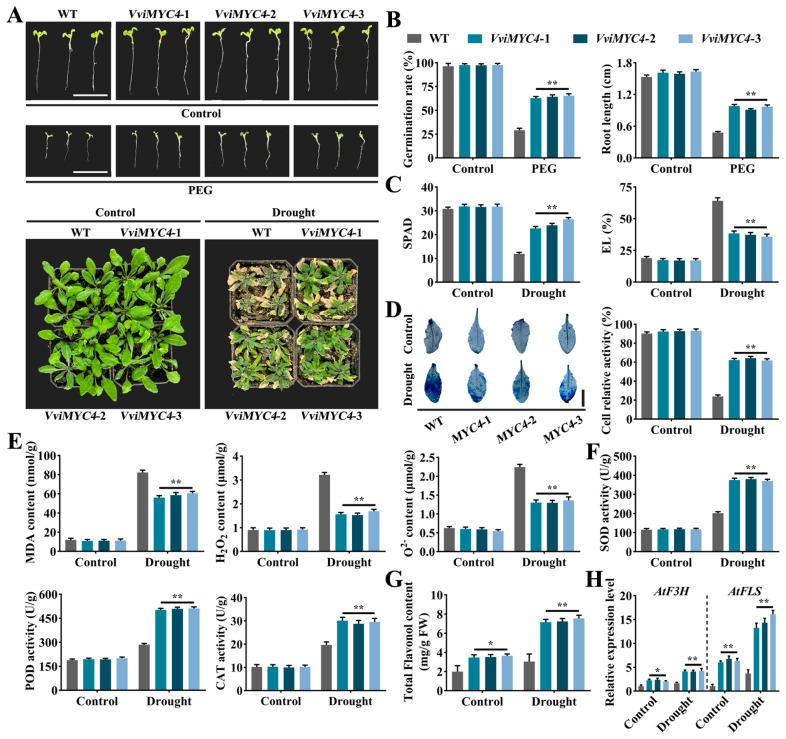
The performance of *VviMYC4* transgenic *A. thaliana* under drought stress. (**A**) Phenotypes of the wild-type and *VviMYC4* transgenic *A. thaliana* seedlings and adult plants under normal and drought conditions. (**B**) The germination rate and root length of the wild-type and *VviMYC4* transgenic *A. thaliana* seedlings under normal and drought conditions. (**C**) The relative chlorophyll content (SPAD value) and electrolyte leakage (EL) in the leaves of the wild-type and *VviMYC4* transgenic *A. thaliana* adult plants under normal and drought conditions. (**D**) Evans blue staining and cell relative activity of the leaves of the wild-type and *VviMYC4* transgenic *A. thaliana* adult plants under normal and drought conditions. (**E**) The contents of malondialdehyde (MDA), hydrogen peroxide (H_2_O_2_), and superoxide anion (O_2_^−^) in the leaves of the wild-type and *VviMYC4* transgenic *A. thaliana* adult plants under normal and drought conditions. (**F**) The activities of superoxide dismutase (SOD), peroxidase (POD) and catalase (CAT) in the leaves of the wild-type and *VviMYC4* transgenic *A. thaliana* adult plants under normal and drought conditions. (**G**) Total flavonol content in the leaves of the wild-type and *VviMYC4* transgenic *A. thaliana* adult plants under normal and drought conditions. (**H**) The expression levels of the flavonol biosynthesis-related genes *AtF3H* and *AtFLS* in the leaves of the wild-type and *VviMYC4* transgenic *A. thaliana* adult plants under normal and drought conditions. Asterisks indicate the significant differences between the different transgenic lines and wild-type lines (* *p* ≤ 0.05, ** *p* ≤ 0.01).

Under normal conditions, no matter in seedlings or adult plants, there were no obvious differences between the wild-type (WT) and *VviMYC4*-overexpressed (OE) lines of *A. thaliana*. The drought-induced phenotypic changes revealed that compared with the withering and wilting state of the wild-type *A. thaliana* seedlings and the yellowing and shedding phenomena of the adult plant leaves, both the *VviMYC4* transgenic seedlings and adult plants exhibited superior growth characteristics, indicating that heterologous overexpression of the *VviMYC4* gene can significantly alleviate the damage caused by drought stress to *A. thaliana* (Figure 5A). Under normal conditions, there were no obvious differences in the physiological and biochemical indexes between the WT and *VviMYC4* transgenic plants. In the face of drought stress, the *VviMYC4* transgenic seedlings demonstrated a remarkable boost in the germination rates and root length when compared to the WT. As for the leaves of the *VviMYC4* transgenic adult plants, there was a notable rise in the SPAD (Soil and Plant Analyzer Development) value, a measure of the relative chlorophyll content, while the electrolyte leakage (EL) decreased, showcasing a considerable enhancement in their ability to withstand drought conditions (Figure 5B,C). The Evans blue cell activity staining revealed no discernible variance between the WT and overexpressing plant leaves under control conditions. When subjected to drought stress, the OE plants exhibited noticeably fainter staining intensity alongside substantially greater cell viability compared to the WT (Figure 5D). Concurrently, drought stress induced a clear reduction in the oxidative stress markers, including malondialdehyde (MDA), hydrogen peroxide (H_2_O_2_), and superoxide anion (O_2_^−^), in the leaves of *VviMYC4* transgenic plants compared to the WT (Figure 5E). Conversely, these transgenic plants showed a pronounced boost in key antioxidant enzyme activities, particularly superoxide dismutase (SOD), peroxidase (POD), and catalase (CAT) (Figure 5F). Further analysis of the total flavonol content in the leaves of the *A. thaliana* adult plants found that compared with the WT, the flavonol level in the leaves of the *VviMYC4* transgenic *A. thaliana* was sensibly increased, and this was even more obvious under drought stress (Figure 5G). Consistent with this, as a positive regulator, *VviMYC4* significantly increased the expression levels of the key genes *AtF3H* and *AtFLS* involved in flavonol biosynthesis in *A. thaliana* (Figure 5H).

### 2.5. Functional Validation of VviMYC4 Gene Through Transient Overexpression in Tobacco Leaves

**Figure 6:**
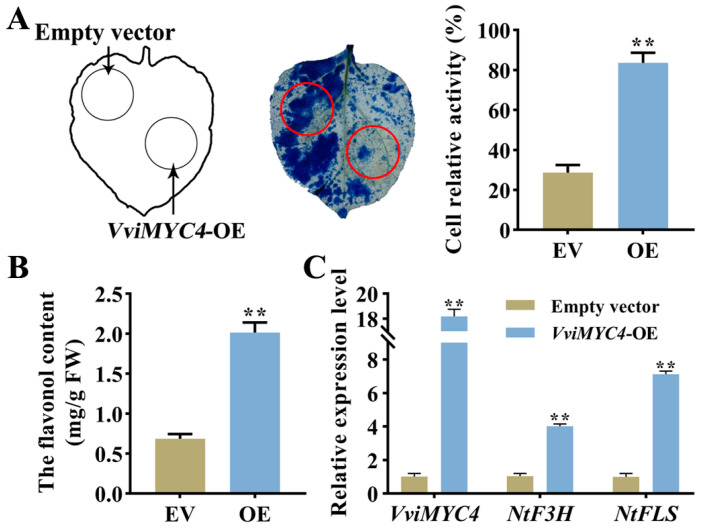
The performance of tobacco leaves with transient overexpression of the empty vector (EV) and *VviMYC4* under drought stress. (**A**) Phenotype and cell relative activity of the EV and *VviMYC4*-overexpressed (OE) tobacco leaves under drought stress. The red circles represent the injection sites of EV and *VviMYC4*-OE on tobacco leaves, respectively. (**B**) Total flavonol content in the EV and *VviMYC4*-OE tobacco leaves under drought stress. (**C**) The expression levels of the flavonol biosynthesis-related genes *NtF3H* and *NtFLS* in the EV and *VviMYC4*-OE tobacco leaves under drought stress. ** indicate the significant differences between *VviMYC4*-OE and EV (*p* ≤ 0.01).

*VviMYC4* was transiently overexpressed in tobacco leaves to verify the gene function under drought stress. The results of the Evans blue staining and cell relative activity analysis revealed that the *VviMYC4*-OE tobacco leaves exhibited markedly greater drought tolerance compared to those injected with an empty vector (EV). This was evidenced by significantly less staining and higher relative cell activity under drought stress (Figure 6A). Furthermore, a substantial increase in the total flavonol content was observed in the *VviMYC4*-OE leaves (Figure 6B). The RT-qPCR results further demonstrated that *VviMYC4* was highly expressed in the overexpressed leaves. Notably, the expression levels of the key flavonol biosynthesis genes, *NtF3H* and *NtFLS*, were strongly induced in the *VviMYC4*-OE leaves, aligning with the changes in the flavonol content (Figure 6C).

### 2.6. Functional Validation of VviMYC4 Gene Through Transient Overexpression in Grapevine Leaves

**Figure 7:**
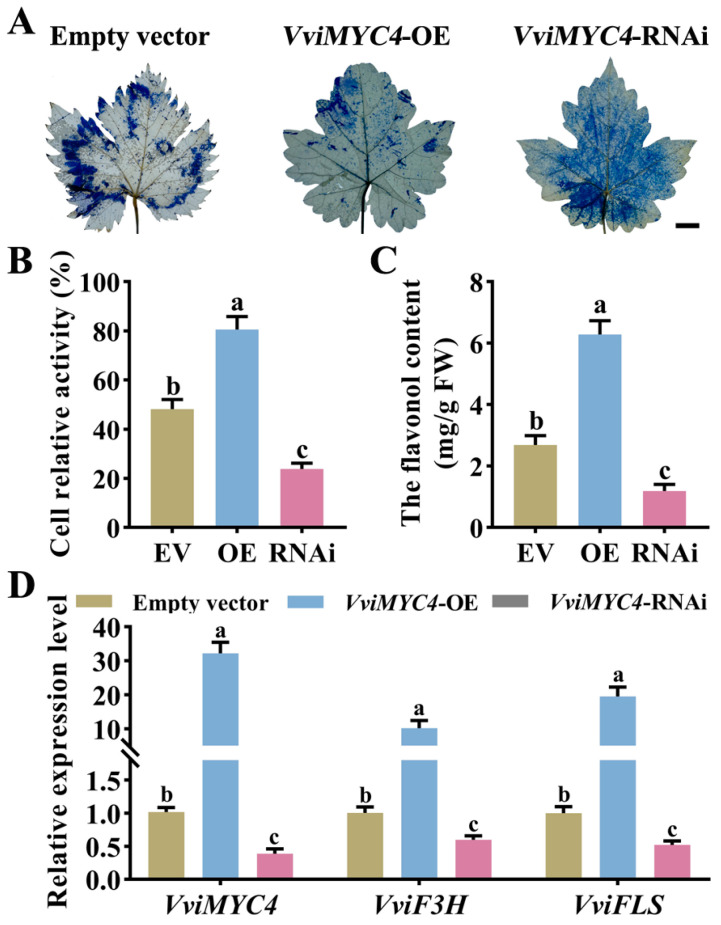
The performance of the transient transformation of grapevine leaves by EV and *VviMYC4* under drought stress. (**A**) Phenotype of the EV, *VviMYC4*-OE and *VviMYC4*-RNAi ‘Cabernet Sauvignon’ grapevine leaves under drought stress. (**B**) The cell relative activity in the EV, *VviMYC4*-OE and *VviMYC4*-RNAi grapevine leaves under drought stress. (**C**) Total flavonol content in the EV, *VviMYC4*-OE and *VviMYC4*-RNAi grapevine leaves under drought stress. (**D**) The expression levels of the flavonol-biosynthesis-related genes *VviF3H* and *VviFLS* in the EV, *VviMYC4*-OE and *VviMYC4*-RNAi grapevine leaves under drought stress. Different lowercase letters indicate the significance of the differences between samples (*p* ≤ 0.05).

The *VviMYC4* gene function was further verified by transiently transforming ‘Cabernet Sauvignon’ grapevine leaves with the constructed *VviMYC4*-OE and *VviMYC4*-RNAi vectors. The results of the Evans blue staining and cell activity assessments revealed that the *VviMYC4*-OE grapevine leaves demonstrated markedly enhanced drought tolerance compared to those transformed with an empty vector (EV). Conversely, the *VviMYC4*-RNAi grapevine leaves displayed heightened sensitivity to drought stress (Figure 7A,B). In alignment with expectations, compared to the EV, it is evident that the *VviMYC4*-OE grapevine leaves showcased a notable hike in the total flavonol content, while the *VviMYC4*-RNAi grapevine leaves experienced a significant drop in the total flavonol levels (Figure 7C). The RT-qPCR analysis revealed that the expression levels of the grapevine flavonol-biosynthesis-related genes, *VviF3H* and *VviFLS*, in both the *VviMYC4*-OE and *VviMYC4*-RNAi grapevine leaves displayed a marked correlation with *VviMYC4* (Figure 7D).

### 2.7. Analysis of the Targeted Regulatory Role of VviMYC4 in Relation to the Promoters of the VviF3H and VviFLS Genes

**Figure 8:**
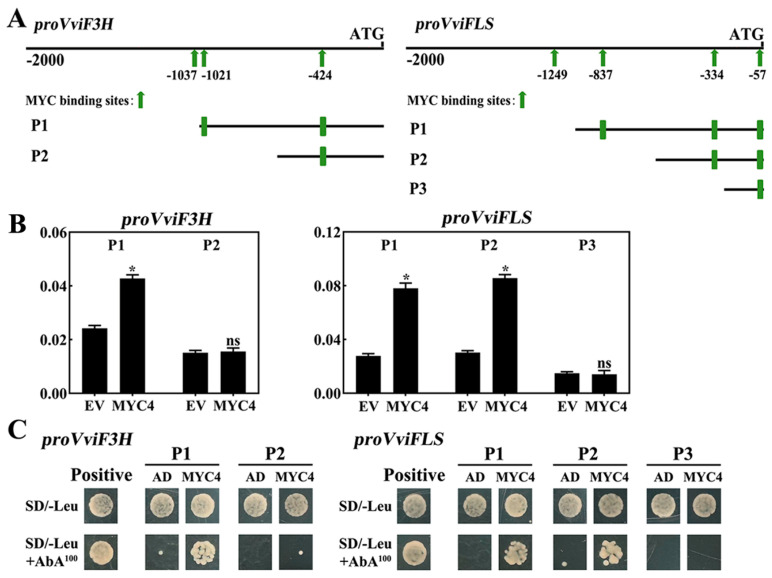
The core binding sites analysis of the *VviF3H* and *VviFLS* promoters regulated by VviMYC4. (**A**) Schematic diagram of the MYC binding sites in the *VviF3H* and *VviFLS* promoters and sequence segmentation according to the sites. (**B**) LUC assays on the regulatory effects of VviMYC4 on the *VviF3H* and *VviFLS* promoters’ segment sequences. * indicate statistically significant differences (*p* ≤ 0.05), ns represents no significant difference. (**C**) Y1H assays verified the binding effect of VviMYC4 on the core regulatory sites of the *VviF3H* and *VviFLS* promoters. pAbAi-P53 and pGADT7-53 were used as positive controls.

The grapevine flavonol biosynthesis genes, *VviF3H* and *VviFLS*, are strongly influenced by the positive regulation of VviMYC4 (Figure 7D). Therefore, there is a need to delve deeper into the core sites where VviMYC4 interacts with the *VviF3H* and *VviFLS* gene promoters. The promoter regions of *VviF3H* and *VviFLS* were isolated from the genomic DNA of the ‘Cabernet Sauvignon’ grapevine, followed by an analysis of the MYC binding sites (E-box elements). The results showed that there were three MYC binding sites (−1037, −1021, and −424 bp) in the promoter of the *VviF3H* gene, and four MYC binding sites (−1249, −837, −334, and −57 bp) in the promoter of the *VviFLS* gene. Consequently, the promoter regions of *VviF3H* and *VviFLS* were truncated, yielding two and three segmented sequences, respectively (Figure 8A). The LUC assay revealed that VviMYC4 exerts a substantial activating effect on the P1 sequence of the *VviF3H* promoter lacking the E-box element at −1037 bp but has no effect on the P2 sequence further lacking the E-box element at −1021 bp. This finding underscores that the E-box element (CAATTG) located at −1021 is the pivotal site through which VviMYC4 modulates the *VviF3H* promoter. Moreover, VviMYC4 significantly activated the P1 and P2 sequences of the *VviFLS* promoter, with the E-box elements at positions −1249 and −837 bp missing, respectively, while it failed to activate the P3 sequence, which lacked the E-box element at −334 bp, indicating that the E-box element (CATGTG) located at −334 bp is essential for VviMYC4 to regulate the *VviFLS* promoter (Figure 8B). The Y1H assay confirmed the critical functional sites, revealing that VviMYC4 lost its binding effect on the *VviF3H* promoter sequence when the E-box site at −1021 bp was deleted, as well as on the *VviFLS* promoter sequence when the E-box site at −334 bp was removed. These findings underscore the significance of these specific E-box elements in mediating VviMYC4’s interaction with both promoters (Figure 8C).

## 3. Discussion

In the response mechanisms of plants to environmental stresses, the regulatory network of gene expression exhibits a high degree of complexity, with transcription factors playing a central regulatory role [24]. These regulatory factors control the expression levels of downstream target genes through multiple molecular mechanisms, including directly binding to DNA sequences or interacting with other regulatory proteins, and ultimately mediate the physiological and biochemical processes by which plants resist adverse stresses [25]. Flavonols, a vital group of flavonoids produced during the secondary metabolism of plants, critically defend plants against environmental stress. They also act as mediators in the interactions between plants and other organisms, thereby helping to maintain the delicate balance of ecosystems [26]. A drought-triggered rise in the flavonol levels in tomatoes [27] and *A. thaliana* [28] suggested that flavonols function as signaling molecules in the drought response.

As crucial regulatory proteins within plants, MYC transcription factors significantly boost the flavonoid biosynthesis pathway and drought stress responses [29]. *IbMYC2* enhances the tolerance to salt and drought stress in sweet potatoes by modulating flavonoid accumulation and ROS scavenging systems [30]. In this study, leveraging the transcriptome data from prior work on the effects of exogenous methyl jasmonate on grapevines under drought stress, we isolated and identified an MYC family transcription factor gene, *VviMYC4*, from ‘Cabernet Sauvignon’ grapevines that was significantly upregulated and positively correlated with flavonol accumulation, and we provided new insights into its expression pattern and functions under drought stress. Sequence analysis revealed that the protein sequence of the *VviMYC4* gene contains a conserved MYC-bHLH_N domain and a bHLH domain at the N-terminal and C-terminal, respectively, belonging to the typical bHLH-MYC transcription factor. Further phylogenetic analysis showed that VviMYC4 has a high degree of homology with grapevine VviMYC2. Previous research has demonstrated that *VviMYC2* can positively regulate flavonol biosynthesis in response to drought stress [21], leading to speculation that *VviMYC4* may have similar functions. An analysis of ‘Cabernet Sauvignon’ grapevine seedling leaf samples revealed that the expression of the *VviMYC4* gene was markedly upregulated under prolonged drought stress conditions. The gene’s transcriptional activity peaked at the 12 h mark following the onset of stress, indicating that *VviMYC4* directly responds to drought signals and operates as an essential mediator in grapevines’ drought-resistant physiological regulatory system. These findings offer valuable perspectives for future investigations into the gene’s function.

Genetic transformation is a crucial approach for boosting plant biomass or resilience against adverse conditions by conferring favorable genetic traits, and it has been widely applied in the characterization of plant gene function [31]. As such, we conducted a functional analysis of the *VviMYC4* gene using a genetic transformation system established in grape suspension cells and the model plant *A. thaliana*. The GFP fluorescence of *VviMYC4* in grape suspension cells indicated its nuclear localization, suggesting that it functions as a transcription factor in the nucleus. Overexpression of the *VviMYC4* gene in grape suspension cells markedly boosted their resilience against drought stress, whereas interfering with the *VviMYC4* expression heightened their vulnerability to such conditions. This evidence underscores *VviMYC4*’s vital function in positively regulating grapevine drought tolerance. In the case of *VviMYC4* transgenic *A. thaliana*, the levels of H_2_O_2_ and O_2_^−^ were significantly reduced, while the activities of antioxidant enzymes were notably increased, leading to enhanced drought tolerance. Notably, the flavonol content and the expression levels of related structural genes aligned with drought resistance, indicating that *VviMYC4* improves antioxidant capacity and ROS scavenging ability by promoting flavonol biosynthesis, thereby enhancing the drought tolerance of transgenic *A. thaliana* plants. These findings are consistent with the regulatory mechanism in *ScTT8* (bHLH) from *Simmondsia chinensis* that enhances cold hardiness by promoting flavonol accumulation and increasing antioxidant activity [32]. Interestingly, tomato SlMYC2 lowers the flavonol levels by suppressing *SlCHS1* expression, modulating ROS-driven stomatal behavior during drought stress [33]. These results highlight that the MYC TFs exhibit functional heterogeneity in the regulation of flavonoid metabolism under drought stress. This includes both positive regulatory mechanisms that boost the expression of structural genes to enhance flavonol biosynthesis and accumulation, and negative regulatory effects that suppress downstream gene expression leading to a decrease in flavonol levels. These multi-level molecular regulatory networks offer fresh perspectives for elucidating the molecular mechanisms of the plant response to drought stress.

The transient expression system serves as a powerful tool for swiftly dissecting gene function, enabling the rapid overexpression or suppression of target genes within plant cells [34]. Known for its simplicity, speed, efficiency, and precision, this approach is invaluable for early-stage assessment of gene functionality and the anticipated phenotypes in transgenic plants [35]. In recent years, transient transformation systems have been successfully established in plants such as grapevines [36], strawberries [37], cassavas [38] and carnations [39] and applied to leaves, fruits, petals, and so on. In this study, we conducted a thorough investigation into the role of the *VviMYC4* gene in mediating flavonol biosynthesis to enhance drought tolerance using transient transformation systems derived from both tobacco and grapevine leaves. It is particularly noteworthy that in grapevine leaves with transient transformation of the *VviMYC4* gene, the expression levels of the key structural genes *VviF3H* and *VviFLS* involved in flavonol synthesis are significantly positively induced by VviMYC4, demonstrating a consistent expression pattern. These findings lay the groundwork for an in-depth analysis of *VviMYC4*’s functions and its underlying mechanisms.

The regulatory mechanism of flavonol metabolism is mainly manifested at the transcriptional level. The core lies in the specific binding of transcription factors to the promoter region of structural genes, thereby directly regulating the expression of key enzyme genes in the flavonol biosynthesis pathway [40]. In this process, the activation mechanism of the transcription factors on the core functional sites of the gene promoters has always been an important focus of the research on metabolic regulation [41]. The bHLH transcription factors regulate downstream genes by specifically recognizing and binding to E-box cis-acting elements (CANNTG) [42]. In this study, we employed bioinformatics approaches to segment the promoter regions of *VviF3H* and *VviFLS* into functional fragments, with a particular focus on pinpointing the bHLH binding sites. Key regulatory sites were then screened and identified through Y1H and LUC assays. This finding highlights the central role of *VviMYC4* in bolstering grapevine drought resistance by specifically activating the expression of *VviF3H* and *VviFLS*, thereby mediating flavonol biosynthesis. Moreover, it provided the crucial experimental evidence for further exploring the functions of related genes and their regulatory networks through targeted site mutation techniques in the follow-up.

Researchers have consistently shown that a defining characteristic of the IIIf-bHLH transcription factor family is the presence of an MYB interaction region (MIR) at their N-terminus [43]. Given the high homology between VviMYC4 and VviMYC2, both phylogenetically grouped in the IIIf subgroup, it stands to reason that VviMYC4 is also capable of binding to MYB TFs. Within plants, bHLH proteins typically partner up with MYB proteins, forming heterodimers to cooperatively regulate the expression of downstream target genes, thus impacting the flavonol metabolism pathway [42]. The C1/LC (MYB/bHLH) complex jointly regulates flavonols in maize, and their co-overexpression in tomatoes increases the kaempferol level in the fruit [44]. The synergistic actions of MrMYB5/MrMYB5L and MrbHLH2 underpin the high accumulation of myricetin in *Morella rubra* [45]. Our preliminary study has already confirmed that there is a protein interaction between VviMYC2 and VviMYB24, which is indispensable for the accumulation of drought-responsive flavonols in grapevine seedlings [21]. Building on these findings, there is an urgent need to identify MYB family members that show significant upregulation under drought stress and can interact with VviMYC4. Systematically unraveling the molecular mechanisms by which these MYBs, alone or in conjunction with VviMYC4, regulate flavonol metabolism is not just a key focus of current research but also paves the way for future in-depth exploration.

In summary, the findings demonstrate that *VviMYC4* could promote flavonol biosynthesis by modulating *VviF3H* and *VviFLS* expression, thereby positively regulating grapevine seedlings’ drought tolerance. This breakthrough not only lays the foundation for in-depth analysis of the multifaceted functions of *VviMYC4* in grapevines but also provides genetic resources to develop drought-tolerant cultivars.

## 4. Materials and Methods

### 4.1. Plant Materials

The one-year-old ‘Cabernet Sauvignon’ grapevine seedlings used in this study were sourced from the Horticultural Station of Shanxi Agricultural University in Taigu (37.429° N, 112.581° E), Shanxi, China. Grapevine seedlings with consistent growth status and 10–12 leaves were selected and evenly divided into two groups. Based on the drought treatment method adopted previously, one group was maintained at 75% relative soil water content (control group) using the weighing method, while the other was kept at 45–50% soil water content (moderate drought) for 7 days (d) (drought group) [21]. Each treatment consisted of three biological replicates. Mature grapevine leaves from the control group (0 h) and those subjected to continuous drought treatment for 3, 6, 12, and 72 h were collected for gene expression analysis. The samples were immediately frozen in liquid nitrogen and kept at −80 °C prior to analysis. Each trial involved three biological replicates.

### 4.2. Gene Cloning and Sequence Analysis

Isolation of the total RNA and cDNA synthesis from ‘Cabernet Sauvignon’ grapevine leaves were performed according to previous research [46]. The primer for the *VviMYC4* gene cloning was designed with Primer Premier 5.0 software (Table S1). Following PCR amplification and ligation to a vector, and after transformation into *E. coli* DH5α (Zomanbio, Beijing, China), the positive clones were sent to Sangon Biotech (Shanghai, China) for sequencing.

The conserved domain was analyzed using CDD in the NCBI database (accessed on [10 June 2024], http://www.ncbi.nlm.nih.gov/Structure/cdd/wrpsb.cgi). Phylogenetic tree and multiple sequence alignment analyses were performed using the MEGA-X software (accessed on [10 June 2024], https://www.megasoftware.net/) and DNAMAN 8 software (accessed on [10 June 2024], https://www.lynnon.com/).

### 4.3. Gene Expression Analysis

Real-time quantitative PCR (RT-qPCR) was employed for assessing the relative expression levels of various genes across distinct samples. The primers are presented in Table S1. The RT-qPCR experiment was carried out using the ChamQ Universal SYBR qPCR Master Mix kit (Vazyme, Nanjing, China) on an ABI Q5 real-time quantitative PCR detection system (Applied Biosystems, Foster City, CA, USA), with the reaction system following the description by Zhao et al. [47]. The expression level of the target gene was examined, with *AtActin*, *NtActin* and *VviUbiquitin* serving as the housekeeping genes, using the 2^−ΔΔCt^ method [48].

### 4.4. Evans Blue Staining

The Evans blue staining method was referred to a previous research report [21]. Briefly, the treated leaves were stained with 0.25% (*w*/*v*) Evans blue for 24 h and then rinsed with water. The method for detecting the cell relative activity was described by Chen et al. [49].

### 4.5. Vector Construction and Plant Transformation

The open reading frame (ORF) of *VviMYC4* was seamlessly integrated into the pCAMBIA2300-GFP plant expression vector. Additionally, both sense and antisense fragments targeting the non-conserved structural domain within the ORF region of *VviMYC4* were successfully ligated into the pFGC5941-RNAi vector. The primers are presented in Table S1.

The transformation of grape suspension cells was carried out in accordance with the procedure detailed by Liu et al. [50], and positive lines were pinpointed by observing the GFP fluorescence signals using a TCS-SP8 laser confocal microscope (Leica, Wetzlar, Germany), with an excitation wavelength of 488 nm, an absorption wavelength of 525 nm, and a spectral wavelength of 500 nm (L5 GFP, Leica, Wetzlar, Germany). Grape suspension cells were cultured at 26 °C for 6 days, with the control group maintained under standard conditions and the drought group supplemented with 4% PEG. After 2 h of treatment, all the suspended cells were removed and dried to determine the fresh weight and for Evans blue staining. Subsequently, the cell staining status was observed under a microscope, and the relative cell activity was measured.

The overexpression vector was introduced into *A. thaliana* via the floral dip method, followed by the selection of positive transgenic lines through PCR amplification and gene expression analysis, as previously outlined [21]. Subsequently, seedlings and adult stages of the wild-type (WT) and T_3_ stable homozygous transgenic lines were treated with 10% PEG and natural drought for 7 d, respectively.

*Agrobacterium*-mediated gene overexpression in the leaves of tobacco (*Nicotiana benthamiana*) and one-year-old ‘Cabernet Sauvignon’ grapevine seedlings was performed based on methods described in previous research [51,52]. The grapevine leaf petioles were wrapped in wet cotton and co-incubated with tobacco plants in the dark for 48 h. Subsequently, the tobacco plants were subjected to natural drought treatment for 7 d, while the grapevine leaves were left to air-dry naturally on dry filter paper for 5 d [21].

### 4.6. Physiological and Biochemical Measurements

The SPAD values, electrolyte leakage (EL), malondialdehyde (MDA) concentrations, and total flavonol content in the leaves of the transgenic materials were assessed in accordance with standardized procedures [21]. Additionally, the levels of hydrogen peroxide (H_2_O_2_, catalog number [H_2_O_2_-1-Y]) and superoxide anion (O_2_^−^, catalog number [SA-1-G]), along with the enzymatic activities of superoxide dismutase (SOD, catalog number [SOD-1-Y]), peroxidase (POD, catalog number [POD-1-Y]), and catalase (CAT, catalog number [CAT-1-Y]), were quantified using specialized assay kits (Suzhou Comin Biotechnology, Suzhou, China).

### 4.7. Cis-Acting Elements Analysis of Promoters

The genomic DNA (gDNA) isolated from the leaves of ‘Cabernet Sauvignon’ grapevines was used as the foundation for amplifying the promoter regions, specifically the 2000 base pairs upstream of the start codon for the *VviF3H* and *VviFLS* genes. The primers are presented in Table S1. The PlantCARE database (accessed on [10 June 2024], http://bioinformatics.psb.ugent.be/webtools/plantcare/html/) was utilized for analyzing the *cis*-acting elements incorporated into these gene promoters.

### 4.8. Dual-Luciferase (LUC) Reporter Assays

The ORF from *VviMYC4* was inserted into the pGreenII 62-SK vector, while the full-length and fragmentary sequences of the *VviF3H* and *VviFLS* promoters were seamlessly cloned into the pGreenII 0800-LUC vector. The primers are presented in Table S1. The resulting recombinant plasmids were then transferred into tobacco leaves using *A. tumefaciens*-induced co-transformation. Following a 48–72 h incubation period, the luminescence was visualized and quantified using a live imaging system (FUSION FX6 EDGE, VILBER, Paris, France), while the LUC/REN ratios were determined with a LUC assay kit (Promega, Madison, WI, USA) [53].

### 4.9. Yeast One-Hybrid (Y1H) Assays

The full-length and sectioned promoter regions of *VviF3H* and *VviFLS* were inserted into the pAbAi vector, and the ORF from *VviMYC4* was inserted into the pGADT7 vector. The primers are presented in Table S1. Subsequently, the fusion vectors containing different promoter sequences were co-transformed with *VviMYC4*-AD into the Y1H Gold yeast strain, respectively. The cultures were incubated at 28 °C for 3 d in SD/-Leu medium, which was supplemented with varying levels of Aureobasidin A (AbA) [54].

### 4.10. Statistical Analysis

The data are shown as the mean ± standard error (SE) obtained from three independent biological replicates. The data were analyzed and visualized by means of SPSS 23.0 (IBM, Armonk, NY, USA) and GraphPad Prism 8.0 (GraphPad Software, San Diego, CA, USA). To establish statistical significance, Student’s *t*-test and a one-way ANOVA with Tukey’s multiple comparison test were used, and *p* ≤ 0.05 was considered significant.

## 5. Conclusions

This study conducted bioinformatics analysis, expression pattern detection, functional characterization, and regulatory mechanism research on grapevine *VviMYC4* and deciphered its role in mediating flavonol biosynthesis in the regulation of grapevine drought stress tolerance. The results indicate that VviMYC4 was highly homologous to VviMYC2, and its expression was significantly induced by drought stress. The transformation of *VviMYC4* in grapevine, *A. thaliana*, and tobacco leaves promoted flavonol accumulation and enhanced their drought tolerance. Additionally, VviMYC4 could specifically bind to the promoters of *VviF3H* and *VviFLS* to activate their expression, thus positively regulating flavonol biosynthesis in grapevine. These findings provide a theoretical and experimental foundation for understanding grapevine drought resistance mechanisms and contribute to molecular breeding strategies aimed at enhancing drought tolerance in grapevines.

## Data Availability

Data are contained within the article or Supplementary Materials.

## Supplementary Materials

The following supporting information can be downloaded at https://www.mdpi.com/article/10.3390/plants14101409/s1. Table S1: Primers used in this study.
