# Supplementary material for: Functional Characterization of Grapevine VviMYC4 in Regulating Drought Tolerance by Mediating Flavonol Biosynthesis
Source: Plants (Basel). 2025 May 8;14(10):1409. doi: 10.3390/plants14101409 (PMC12115097; doi:10.3390/plants14101409)
Supplement: Supplementary file 1 [file plants-14-01409-s001.zip › plants-3584551-supplementary.pdf]

Table S1. Primers used in this study

Primer name	Primer sequence (5′-3′)	Usage
VviMYC4-F	ATGGAAGATATAATCTCTTCGTCTTC	Gene cloning
VviMYC4-R	TTACTGCATTCTTTTGAGTATTGC	
VviMYC4-2300-GFP-F	ACGGGGGACGAGCTCGGTACCATGGAAGATATAATCTCTTCGTCTTC	Constructing gene vectors
VviMYC4-2300-GFP-R	GGTGTCGACTCTAGAGGATCCCTGCATTCTTTTGAGTATTGCAG	
VviMYC4-pFGC5941-F+	ATTACAATTACATTTACAATTACCAAGCCGAGATGTATGATAATCAAAG	
VviMYC4-pFGC5941-R+	GTAACATAAGAAATTCTTACACATTTTCATGGACTCTTCACTCGTCAA	
VviMYC4-pFGC5941-F-	TTTGGTCAATTTGCAGGTATTTGGATCATGGACTCTTCACTCGTCAA	
VviMYC4-pFGC5941-R-	AGTCCCGGGTCTTAATTAACTCTCTAGCCGAGATGTATGATAATCAAAG	
VviMYC4-AD-F	TACGACGTACCAGATTACGCTCATATGATGGAAGATATAATCTCTTCGTCTTC	
VviMYC4-AD-R	TCTACGATTCATCTGCAGCTCGAGTTACTGCATTCTTTTGAGTATTGC	
VviMYC4-SK-F	AGCTCCACCGCGGTGGCGGCCATGGAAGATATAATCTCTTCGTCTTC	
VviMYC4-SK-R	TTCCTGCAGCCCGGGGGATCCTTACTGCATTCTTTTGAGTATTGC	
qVviUbiquitin-F	GCTCGCTGTTTTGCAGTTCTAC	RT-qPCR
qVviUbiquitin-R	AACATAGGTGAGGCCGCACTT	
qVviMYC4-F	AACCTCATGTGGCGTACTGG	
qVviMYC4-R	TGGATGGCTTTGAGCCGAAT	
qVviF3H-F	CAAGGTGGCCTACAACGACT	
qVviF3H-R	GCCGCCAACTTCATCGATTC	
qVviFLS-F	AACCAAGATGACTAAGAACC	
qVviFLS-R	CTTCTGTGACTTCCCTGTAG	

qAtActin-F	GGAACTTGAGAAGGAGCCTAAG	
qAtActin-R	CAACACCAACAGCAACAGTCT	
qAtF3H-F	GGAAGAGATTTGGAGCTTGC	
qAtF3H-R	TCGACAGGCTTGTCAACTTC	
qAtFLS-F	TCACAACATTCCGAGGTCCAA	
qAtFLS-R	CTTCGTCGGGATCGCTTAGA	
qNtActin-F	CTGAGGTCCTTTTCCAACCA	
qNtActin-R	TACCCGGGAACATGGTAGAG	
qNtF3H-F	CAAGGCATGTGTGGATATGG	
qNtF3H-R	TGTGTCGTTTCAGTCCAAGG	
qNtFLS-F	AAGACTCCAGGGTCGCAG	
qNtFLS-R	CTGTAGGAAGGAGGGCTT	
pVviF3H-F	TGGTGGTTGTCGACTTCCAT	
pVviF3H-R	GGACGTCGCCTTCTTTCTCTA	Gene promoter cloning
pVviFLS-F	ATTTTTCTTCAACTTTTGATGCC	
pVviFLS-R	CCTTTTGTCGTTTCACTTGGAT	
pVviF3H-LUC-F	GGGCCCCCCCTCGAGGTCGACTGGTGGTTGTCGACTTCCAT	
pVviF3H-LUC-R	TGTTTTTGGCGTCTTCCATGGGGACGTCGCCTTCTTTCTCTA	
pVviFLS-LUC-F	GGGCCCCCCCTCGAGGTCGACATTTTTCTTCAACTTTTGATGCC	LUC assay
pVviFLS-LUC-R	TGTTTTTGGCGTCTTCCATGGCCTTTTGTCGTTTCACTTGGAT	
pVviF3H-P1-LUC-F	GGGCCCCCCCTCGAGGTCGACCATCGATCAATTGTTTGATCAATC	
pVviF3H-P2-LUC-F	GGGCCCCCCCTCGAGGTCGACGACACTATGAACAAATCCAAAGGA	

pVviFLS-P2-LUC-F	GGGCCCCCCCTCGAGGTCGACAGCGAAATACAGGGTCCACAA	
pVviFLS-P3-LUC-F	GGGCCCCCCCTCGAGGTCGACTTCTGGAAAAGAATGACCAAAAG	
pVviFLS-P4-LUC-F	GGGCCCCCCCTCGAGGTCGACACAGTCTTTATTGGGACGCCC	
pVviF3H-pAbAi-F	AAATGATGAATTGAAAAGCTTTGGTGGTTGTCGACTTCCAT	
pVviF3H-pAbAi-R	ATACAGAGCACATGCCTCGAGGGACGTCGCCTTCTTTCTCTA	
pVviFLS-pAbAi-F	AAATGATGAATTGAAAAGCTTATTTTTCTTCAACTTTTGATGCC	
pVviFLS-pAbAi-R	ATACAGAGCACATGCCTCGAGCCTTTTGTCGTTTCACTTGGAT	
pVviF3H-P1-pAbAi-F	AAATGATGAATTGAAAAGCTTCATCGATCAATTGTTTGATCAATC	Y1H assay
pVviF3H-P2-pAbAi-F	AAATGATGAATTGAAAAGCTTGACACTATGAACAAATCCAAAGGA	
pVviFLS-P1-pAbAi-F	AAATGATGAATTGAAAAGCTTAGCGAAATACAGGGTCCACAA	
pVviFLS-P2-pAbAi-F	AAATGATGAATTGAAAAGCTTTTCTGGAAAAGAATGACCAAAAG	
pVviFLS-P3-pAbAi-F	AAATGATGAATTGAAAAGCTTACAGTCTTTATTGGGACGCCC	
